# Supplementary material for: Gold catalysts containing interstitial carbon atoms boost hydrogenation activity
Source: Nat Commun. 2020 Sep 14;11:4600. doi: 10.1038/s41467-020-18322-x (PMC7490344; doi:10.1038/s41467-020-18322-x)
Supplement: Supplementary file 1 — Supplementary Information [file 41467_2020_18322_MOESM1_ESM.pdf]

Supplementary Information

Gold catalysts containing interstitial carbon atoms boost hydrogenation activity

Sun et al.

Gold catalysts containing interstitial carbon atoms boost hydrogenation activity

Yafei Sun[1†], Yueqiang Cao[2†], Lili Wang[1†], Xiaotong Mu[1], Qingfei Zhao[1], Rui Si[3], Xiaojuan Zhu[1], Shangjun Chen[1], Bingsen Zhang[4], De Chen[5], Ying Wan[1★]

[1]Key Laboratory of Resource Chemistry of Ministry of Education, Shanghai Key Laboratory of Rare Earth Functional Materials, and Department of Chemistry, Shanghai Normal University, Shanghai 200234, China.

[2]State Key Laboratory of Chemical Engineering, East China University of Science and Technology, Shanghai 200237, China.

[3]Shanghai Synchrotron Radiation Facility, Shanghai Institute of Applied Physics, Chinese Academy of Sciences, Shanghai 201204, China.

[4]Shenyang National Laboratory for Materials Science, Institute of Metal Research, Chinese Academy of Sciences, Shenyang 110016, China.

[5]Department of Chemical Engineering, Norwegian University of Science and Technology, Trondheim N-7491, Norway.

[†]These authors contributed equally.

[★]Correspondence should be addressed to Y.W. (email: ywan@shnu.edu.cn)

Contents

Supplementary Figures **4**

Supplementary Tables **27**

Supplementary Methods **36**

Supplementary References **39**

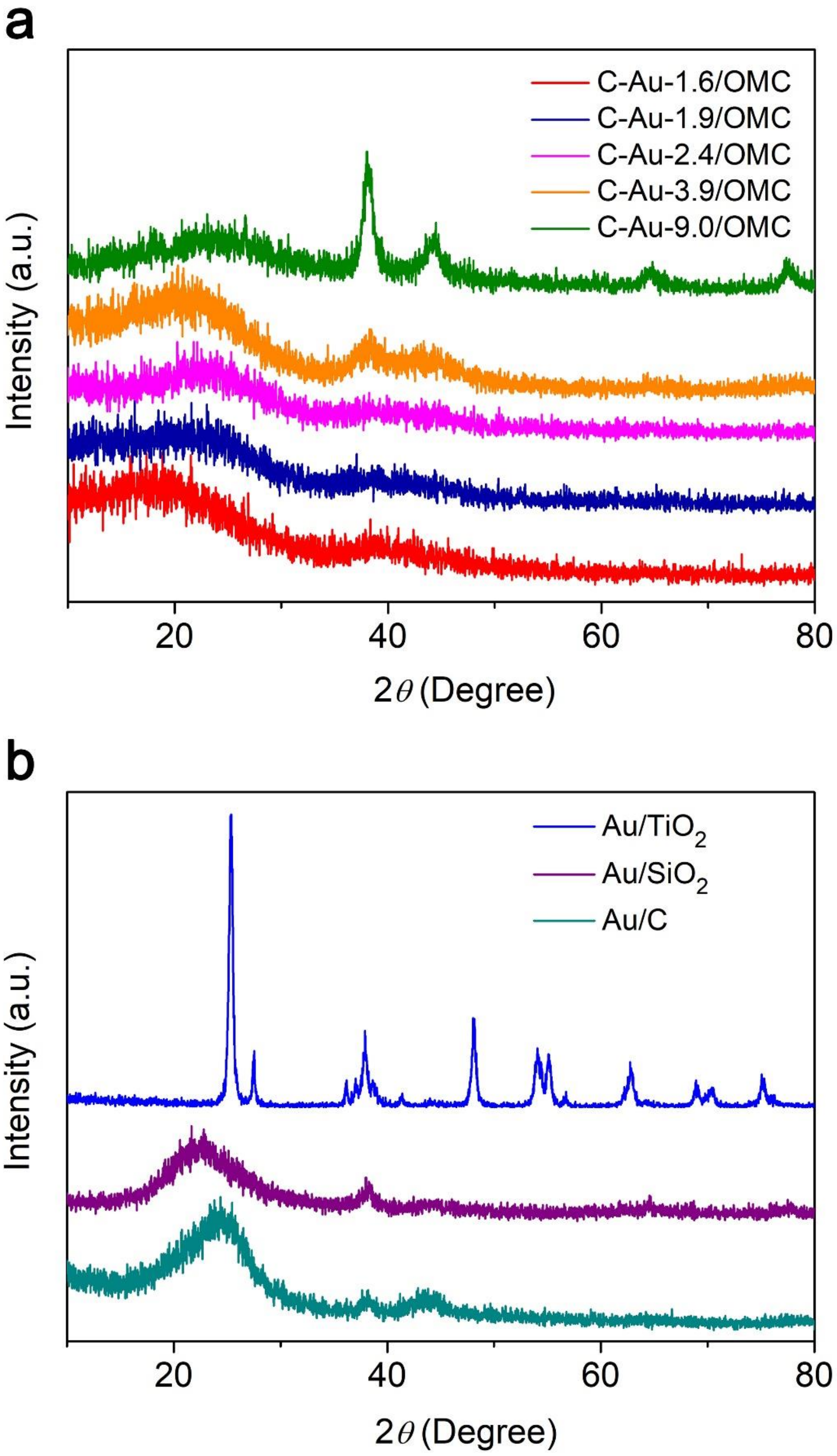

Supplementary Figure 1. Wide-angle X-ray diffraction (WAXRD) patterns for (a) ordered mesoporous carbon-supported Au nanocatalysts with interstitial carbon with different sizes of Au nanoparticles (C-Au-*n*/OMC, where C-Au represents Au with interstitial carbon atoms and *n* denotes the estimated gold nanoparticle size in nanometers.) and (b) commercial Au nanocatalysts supported on titanium, silica and activated carbon.

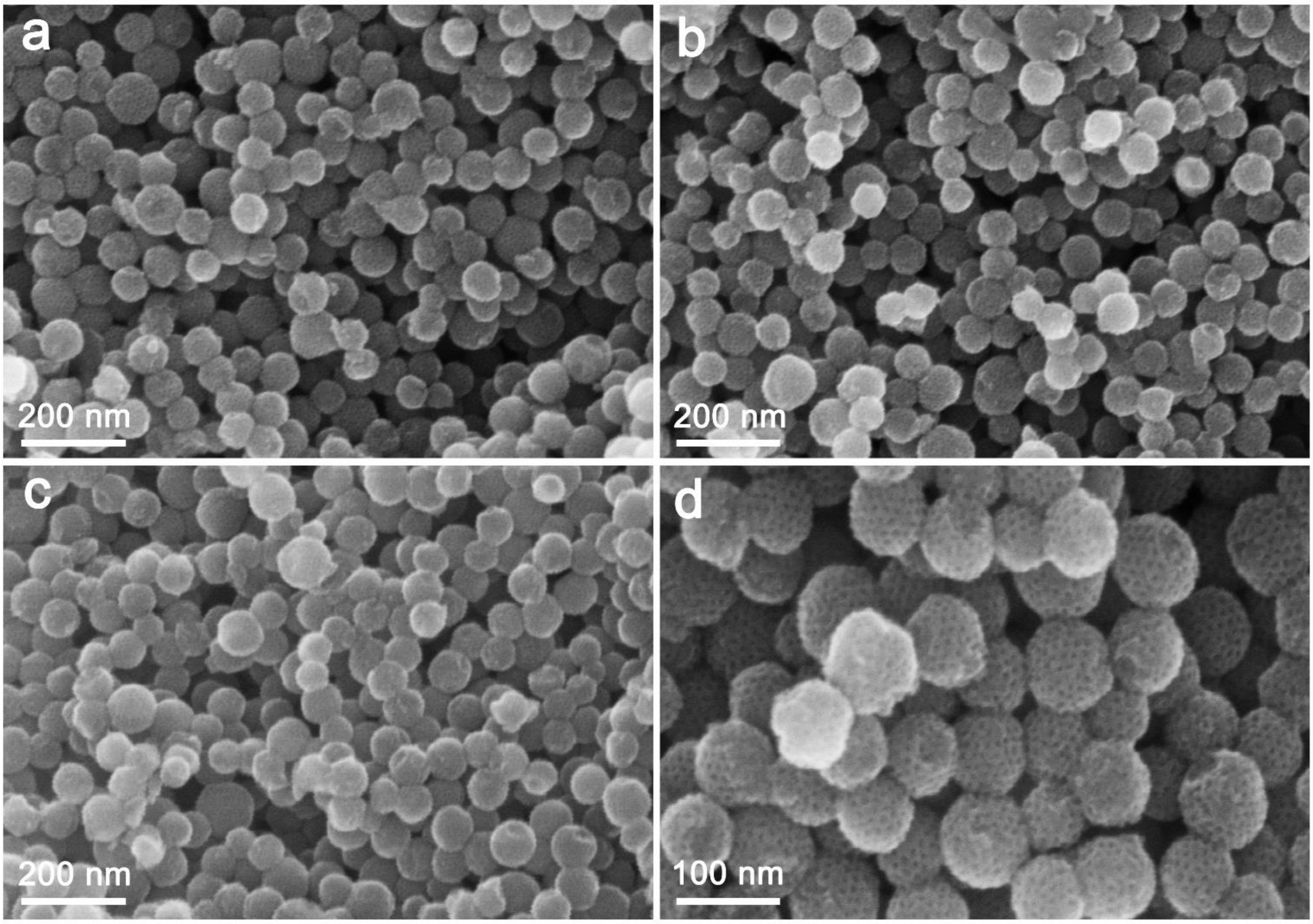

Supplementary Figure 2. High-resolution scanning electron microscope (HRSEM) images of ordered mesoporous carbon-supported C-Au nanocatalysts. (a) C-Au-1.6/OMC, (b) C-Au-1.9/OMC, (c) C-Au-3.9/OMC, and (d) C-Au-9.0/OMC.

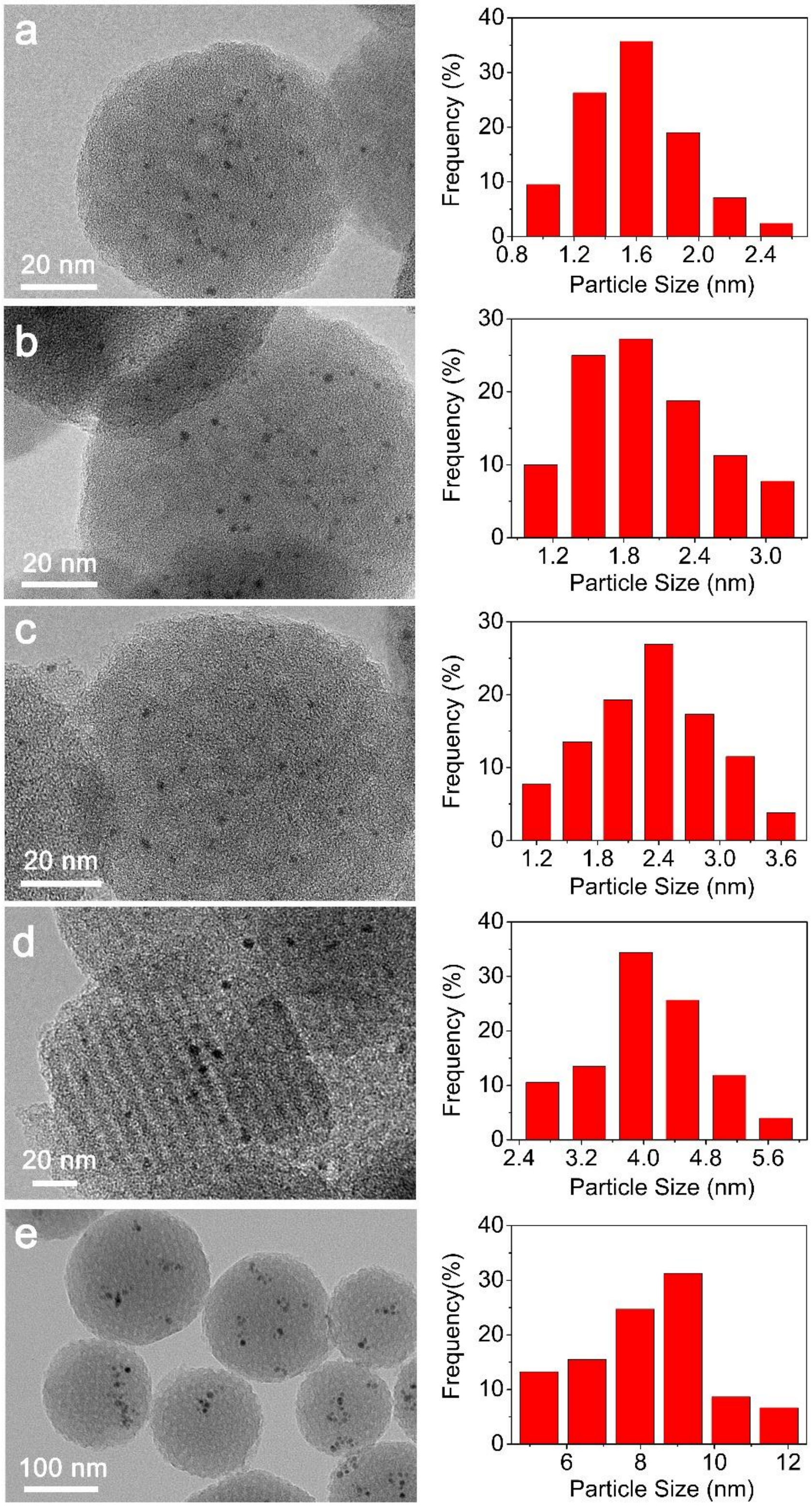

Supplementary Figure 3. Transmission electron microscope (TEM) images and particle size distribution curves obtained from at least 200 nanoparticles for (a) C-Au-1.6/OMC, (b) C-Au-1.9/OMC, (c) C-Au-2.4/OMC, (d) C-Au-3.9/OMC, and (e) C-Au-9.0/OMC.

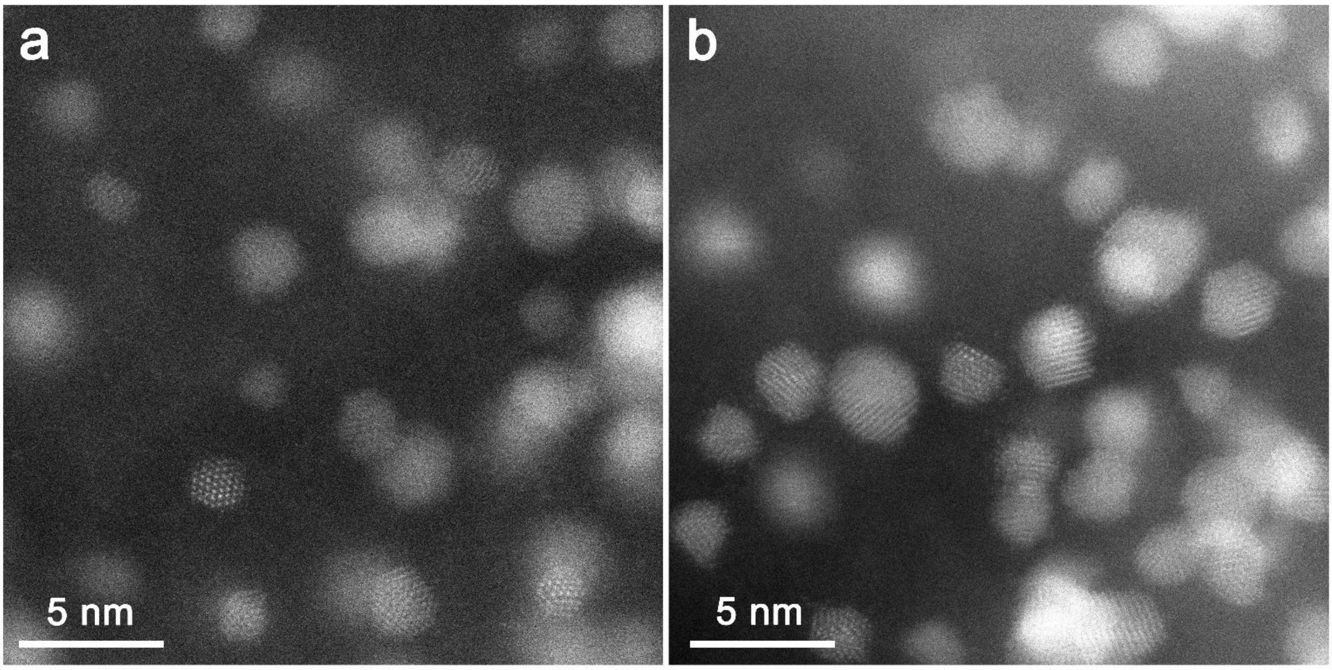

Supplementary Figure 4. High-angle annular dark-field spherical aberration corrected-scanning transmission electron microscope (HAADF-ACSTEM) images for (a) C-Au-1.6/OMC, and (b) C-Au-3.9/OMC.

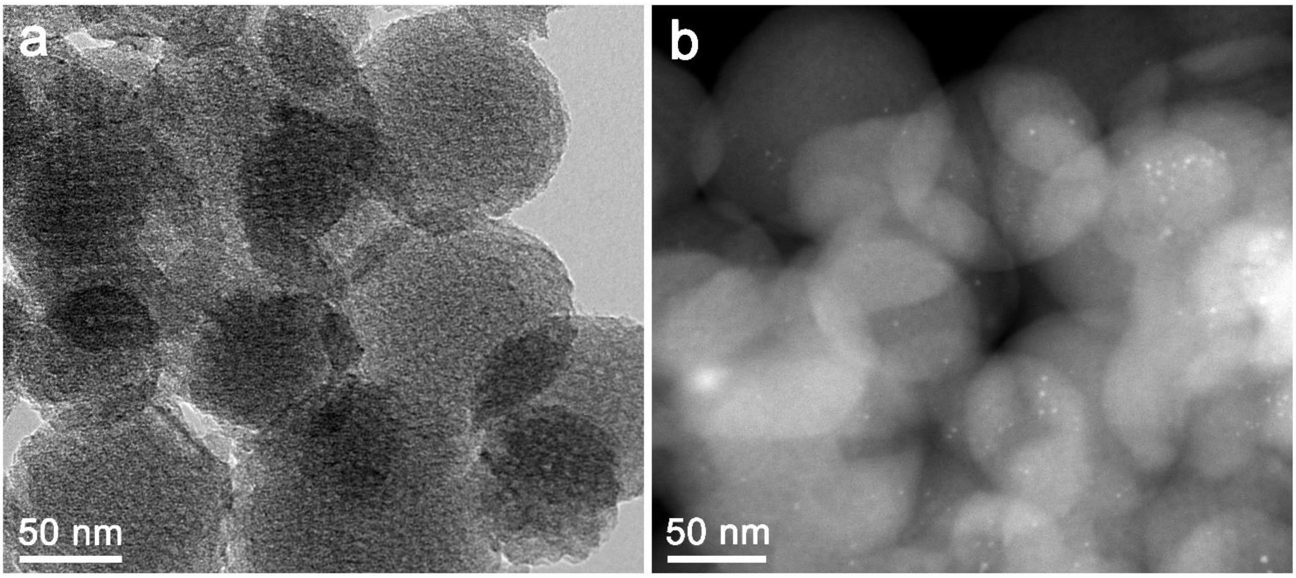

Supplementary Figure 5. (a) TEM and (b) HAADF-STEM images for C-Au-2.4/OMC.

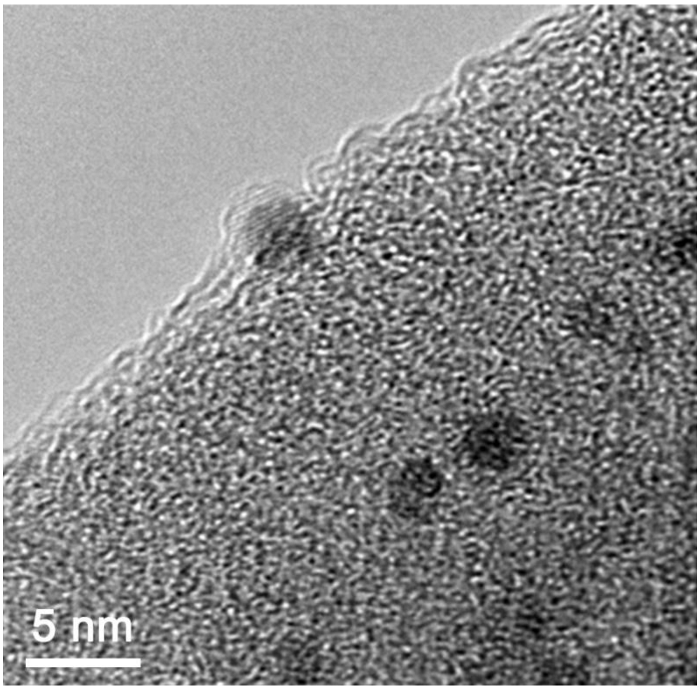

Supplementary Figure 6. TEM image of Au particles covered with graphene caps synthesized in the presence of CH_4 gas.

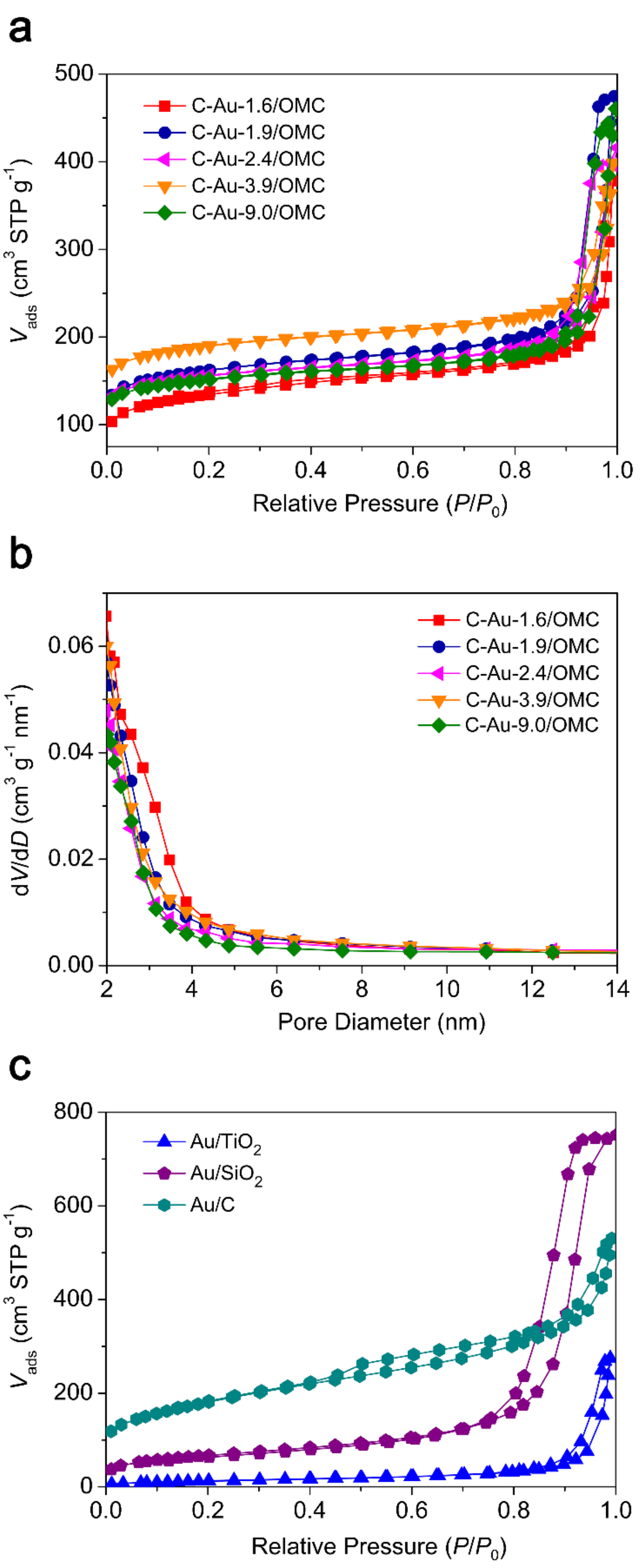

Supplementary Figure 7. Structural properties of Au nanocatalysts. (a, c) N_2 sorption isotherms and (b) pore size distribution curves for (a, b) ordered mesoporous carbon supported C-Au nanocatalysts and (c) commercial Au nanocatalysts supported on titanium, silica and activated carbon.

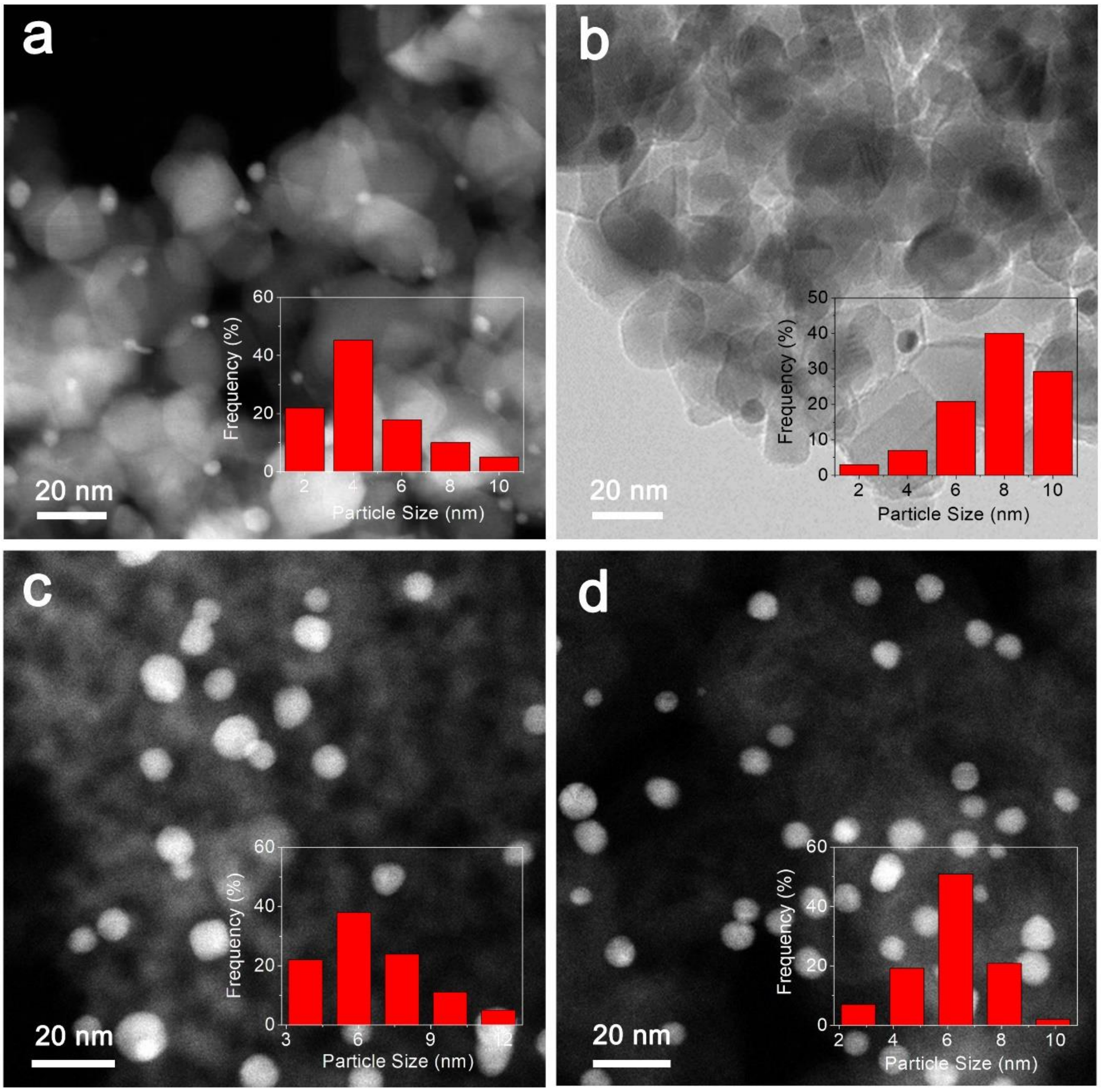

Supplementary Figure 8. (a, c, d) HAADF-STEM images of new commercial Au nanocatalysts supported on titanium, silica and activated carbon. (b) TEM image for reused Au/TiO_2-R. The insets are the particle size distribution curves of the Au nanoparticles measured from at least 200 nanoparticles.

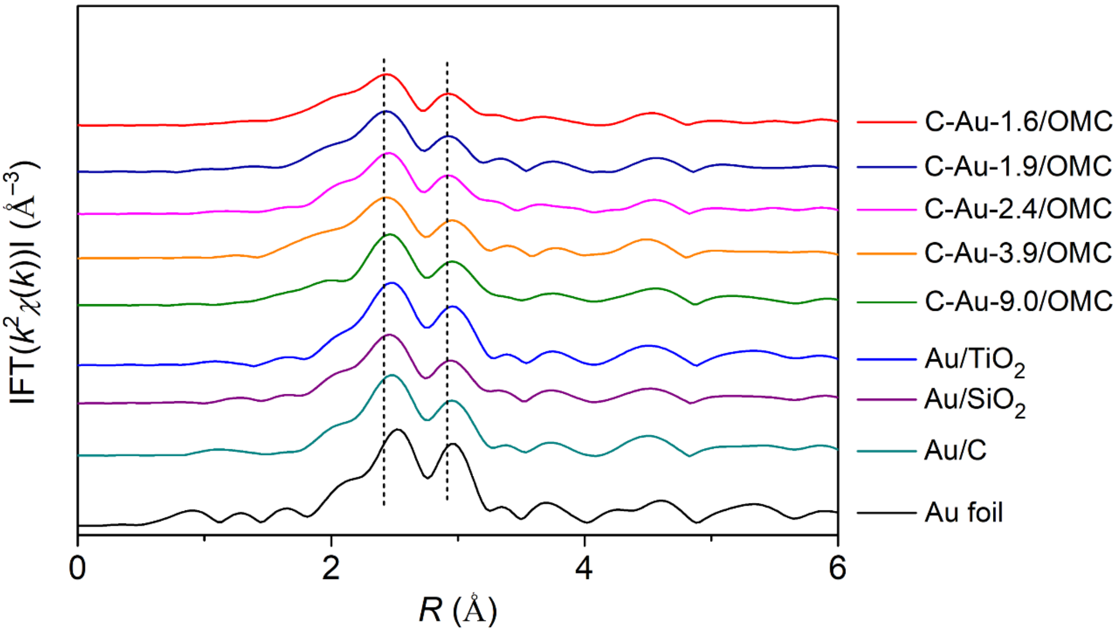

Supplementary Figure 9. k^3-weighted and Fourier transformed magnitudes of the extended X-ray absorption fine structure (EXAFS) spectra (|FT($k^2\chi(k)$)|) of the Au L_3-edge of ordered mesoporous carbon-supported C-Au nanocatalysts. For comparison, the spectra for commercial Au nanocatalysts supported on titanium, silica and activated carbon and Au foil are also given.

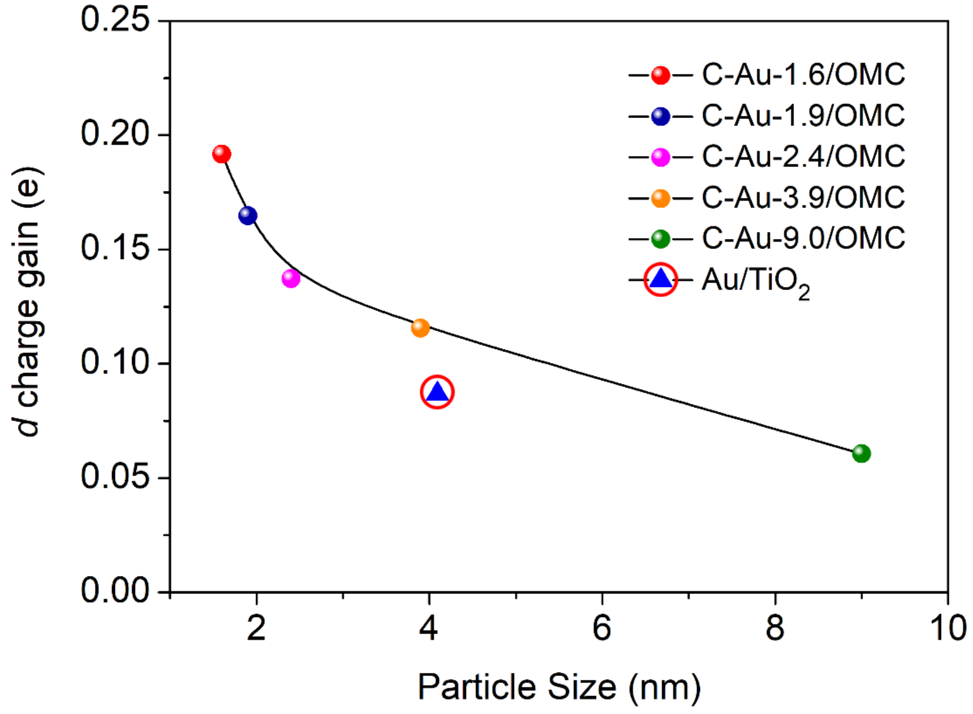

Supplementary Figure 10. Plot of *d* charge gain on C-Au nanocatalysts along with the size of the Au particles. For comparison, the spectrum for a commercial Au nanocatalyst supported on titanium is also given.

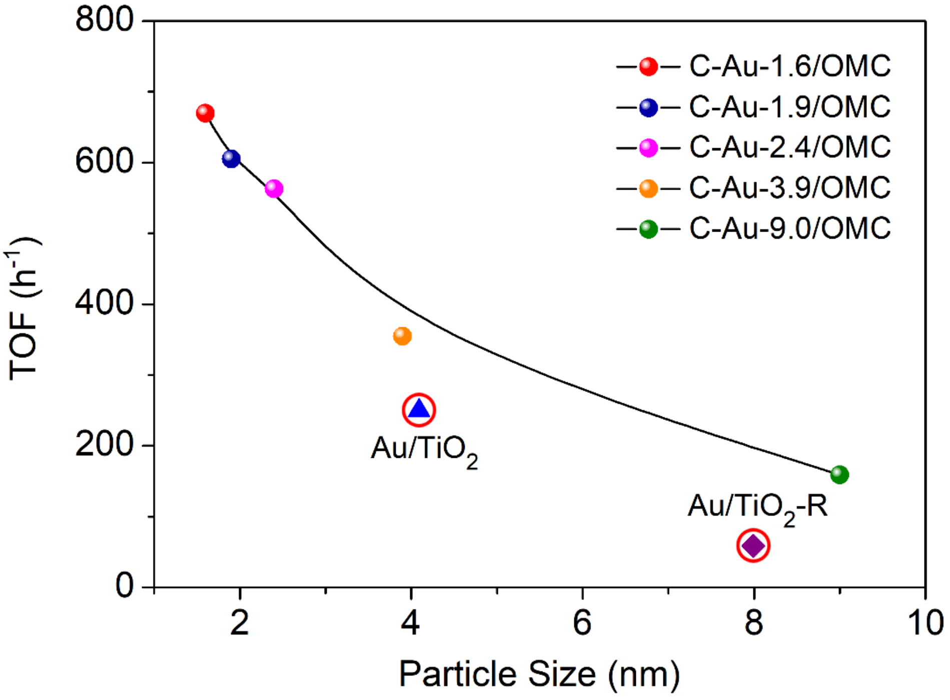

Supplementary Figure 11. Plot of turn-over frequency (TOF) against the size of Au nanoparticles in the selective hydrogenation of 3-nitrostyrene (3-NS) over different Au nanocatalysts. Reaction conditions: 0.78 - 1.87 μmol of Au, 0.41 mmol of 3-NS, 5 mL of ethanol, 140 °C, 4.0 MPa H_2.

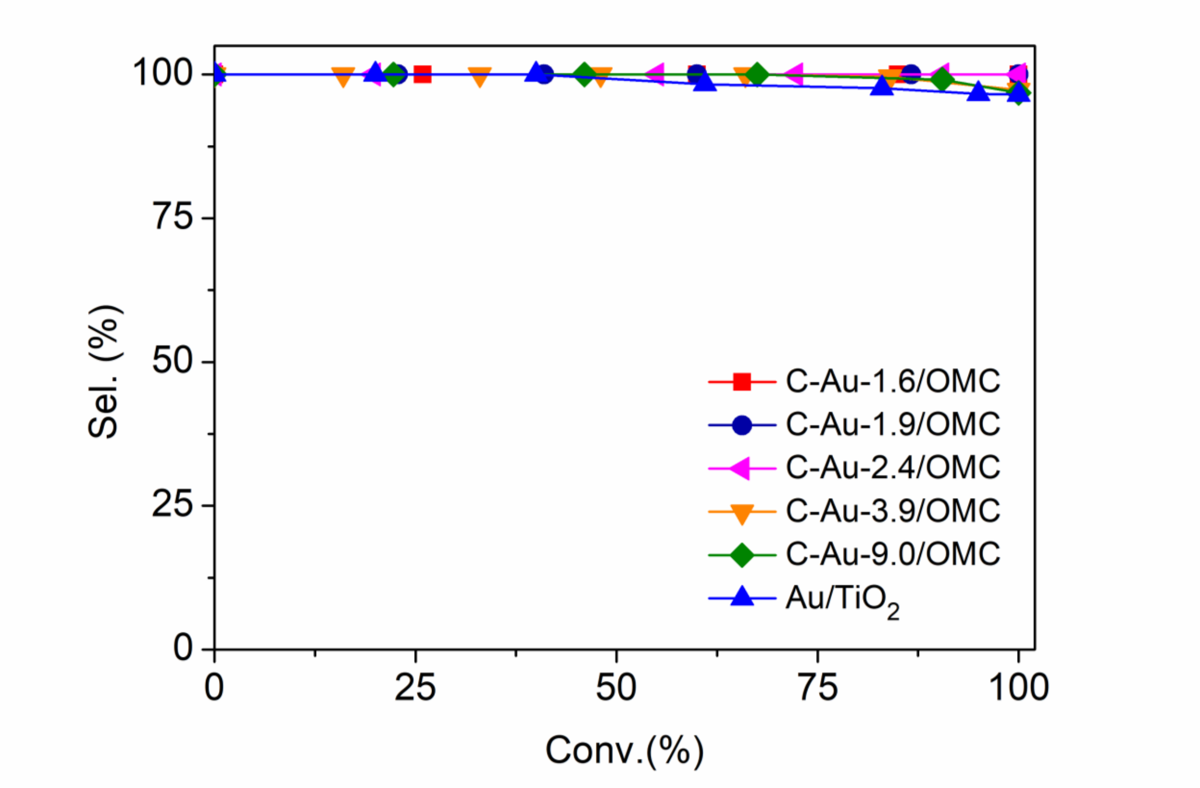

Supplementary Figure 12. Compilation of the selectivity to 3-vinylaniline (3-VA) as a function of the conversion of 3-NS over C-Au nanocatalysts. Reaction conditions: 0.78 - 1.87 μmol of Au, 0.41 mmol of 3-NS, 5 mL of ethanol, 140 °C, 4.0 MPa H_2.

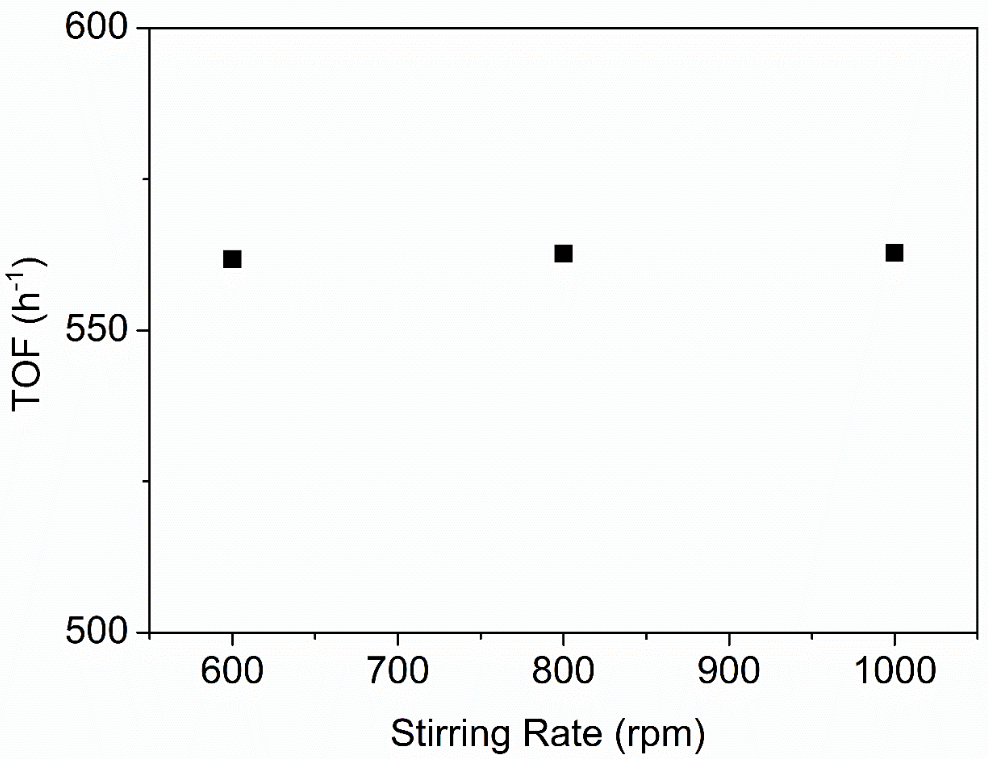

Supplementary Figure 13. Mass transfer limitation tests. Effect of the stirring rate on the TOF of 3-NS hydrogenation using the C-Au-2.4/OMC catalyst. Reaction conditions: 20 mg of the C-Au-2.4/OMC catalyst, 0.41 mmol of 3-NS, 5 mL of ethanol, 140 °C, 4.0 MPa H_2.

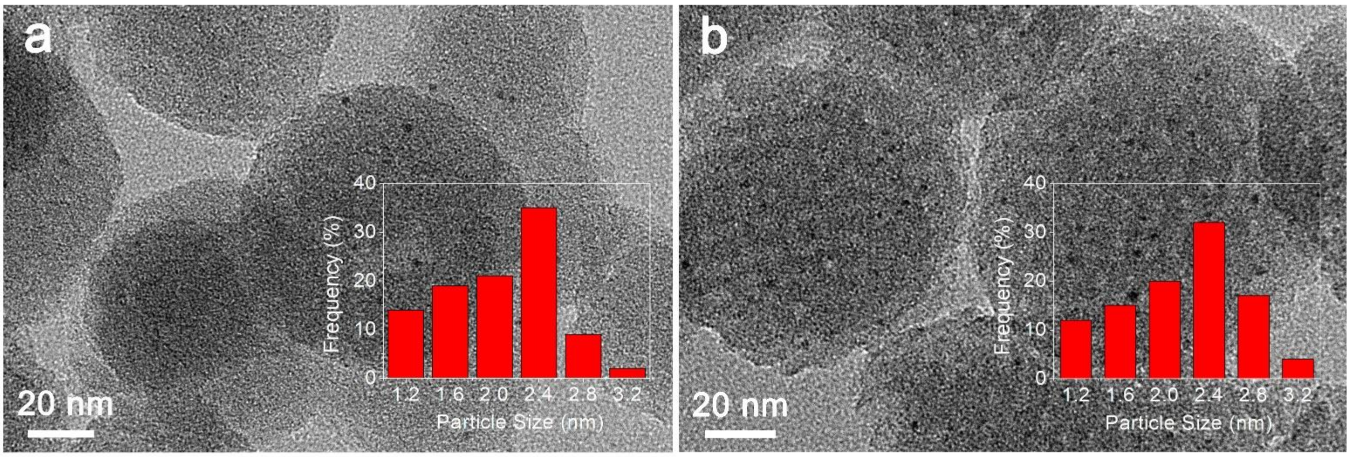

Supplementary Figure 14. TEM images for (a) 0.50 wt% C-Au-2.4/OMC and (b) 0.80 wt% C-Au-2.4/OMC. The insets are particle size distribution curves of the Au nanoparticles measured from at least 200 nanoparticles.

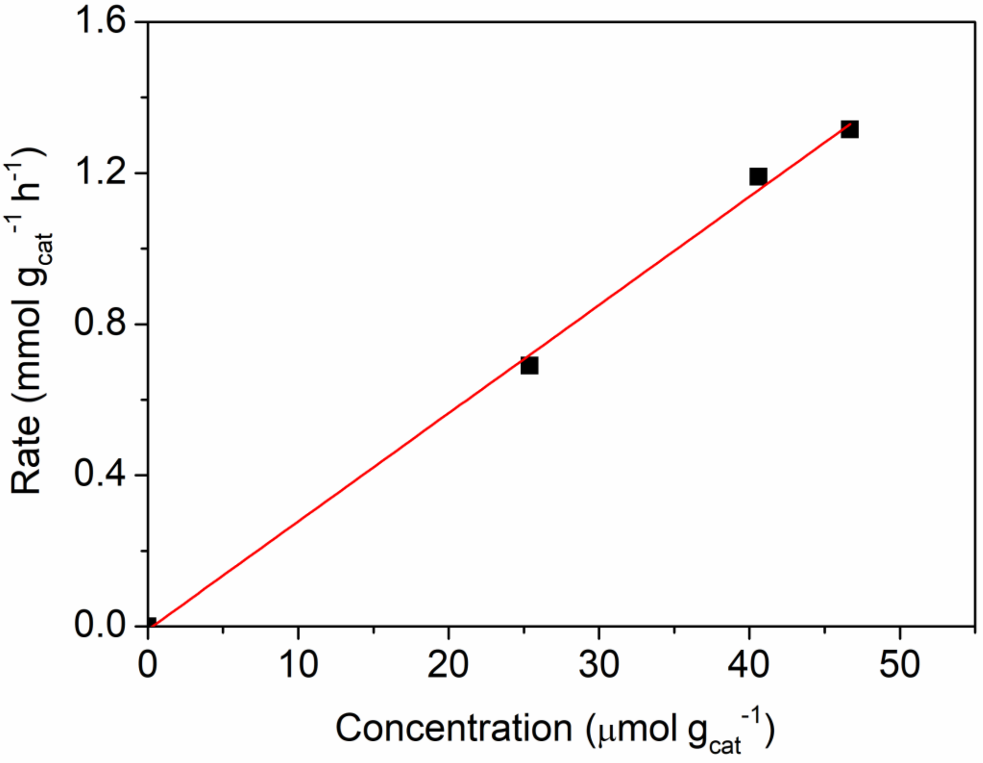

Supplementary Figure 15. Mass transfer limitation tests. Effect of the Au loading (Madon-Boudart test) on the reaction rate of 3-NS hydrogenation over the C-Au-2.4/OMC catalyst with different Au concentrations (0.5 - 1.0 wt%). Reaction conditions: 20 mg of C-Au-2.4/OMC catalyst, 0.41 mmol of 3-NS, 5 mL of ethanol, 140 °C, 4.0 MPa H_2.

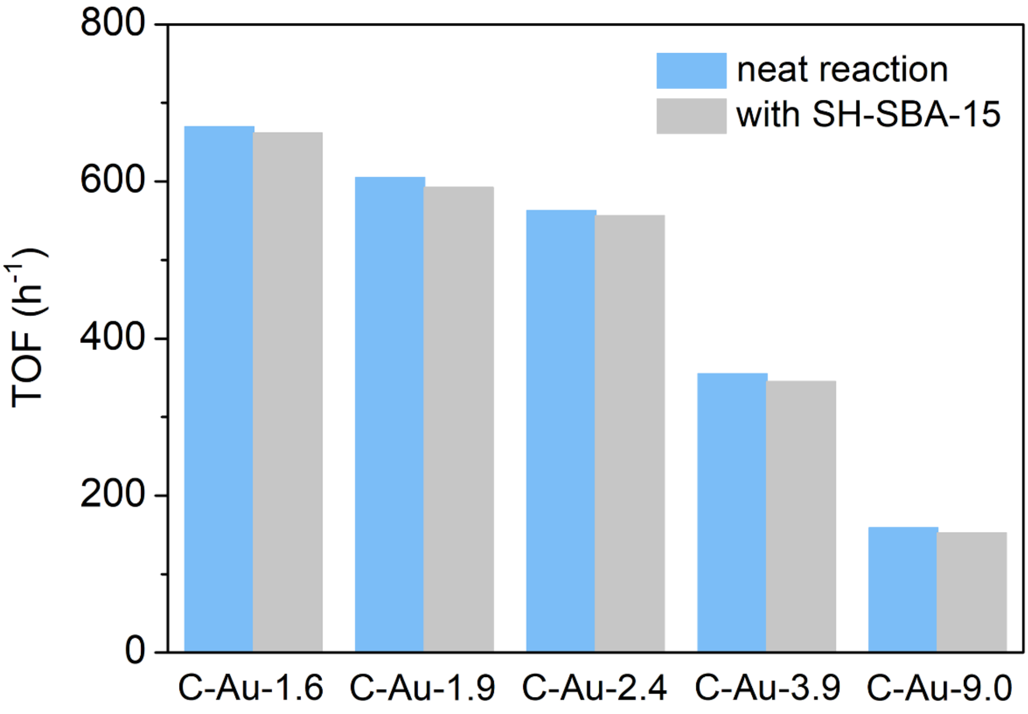

Supplementary Figure 16. Trapping test. Comparison of the conversion of 3-NS in the absence and presence of the solid trapping agent SH-SBA-15 over C-Au nanocatalysts. Reaction conditions: 0.78 - 1.87 μmol of Au, 43.68 - 104.72 mg SH-SBA-15, 0.41 mmol of 3-NS, 5 mL of ethanol, 140 °C, 4.0 MPa H_2.

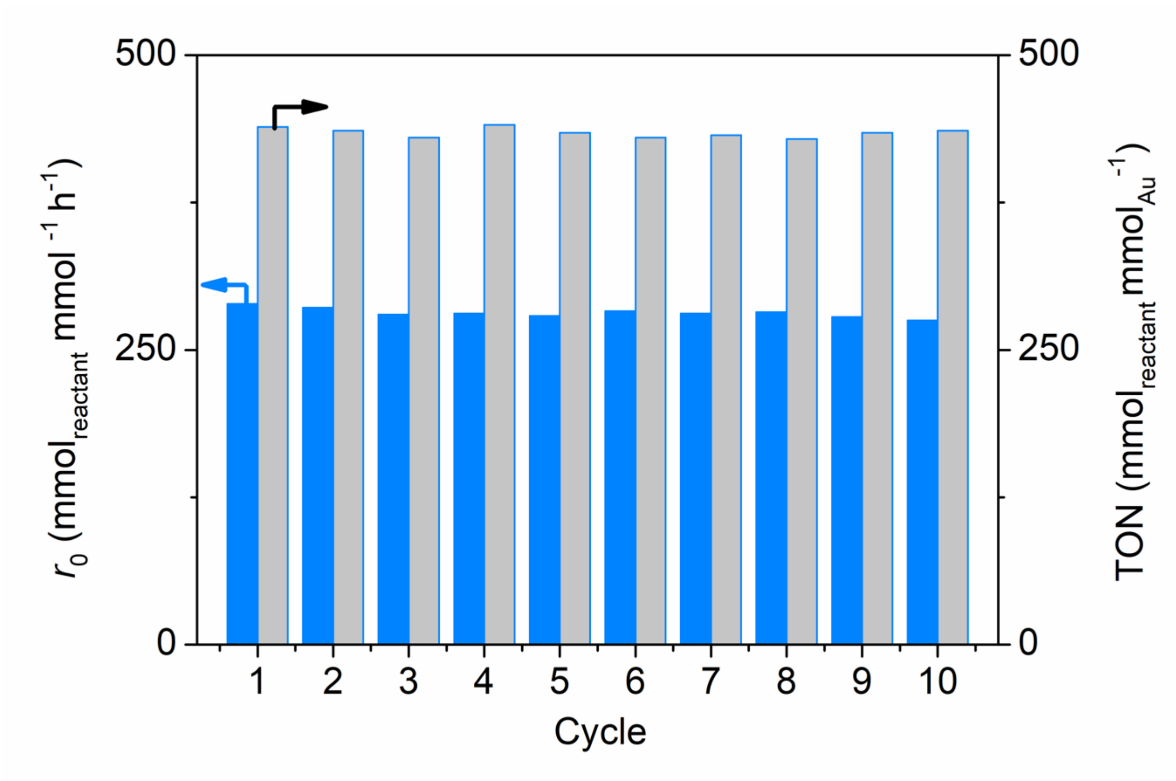

Supplementary Figure 17. Reusability in terms of the initial reaction rate (r_0) and turn over number (TON) in successive runs with the recovered C-Au-2.4/OMC catalyst in the selective hydrogenation of 3-NS. Reaction conditions: 20 mg of C-Au-2.4/OMC, 0.41 mmol of 3-NS, 5 mL of ethanol, 140 °C, 800 rpm, 4.0 MPa H_2.

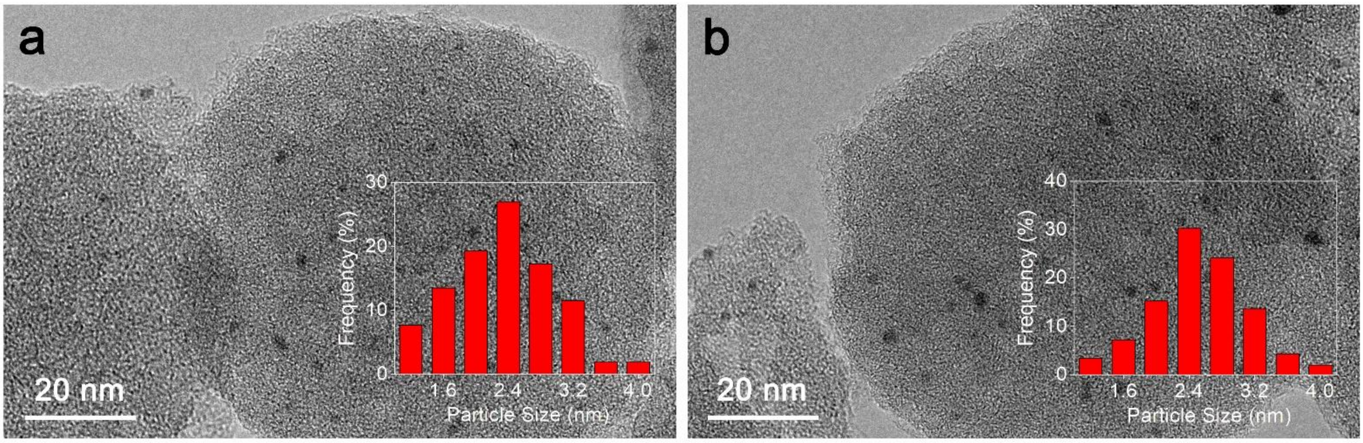

Supplementary Figure 18. TEM images for the (a) fresh C-Au-2.4/OMC catalyst and (b) used C-Au-2.4/OMC-R10 catalyst which is the catalyst after being used in 10 runs. The insets are particle size distribution curves of the Au nanoparticles measured from at least 200 nanoparticles.

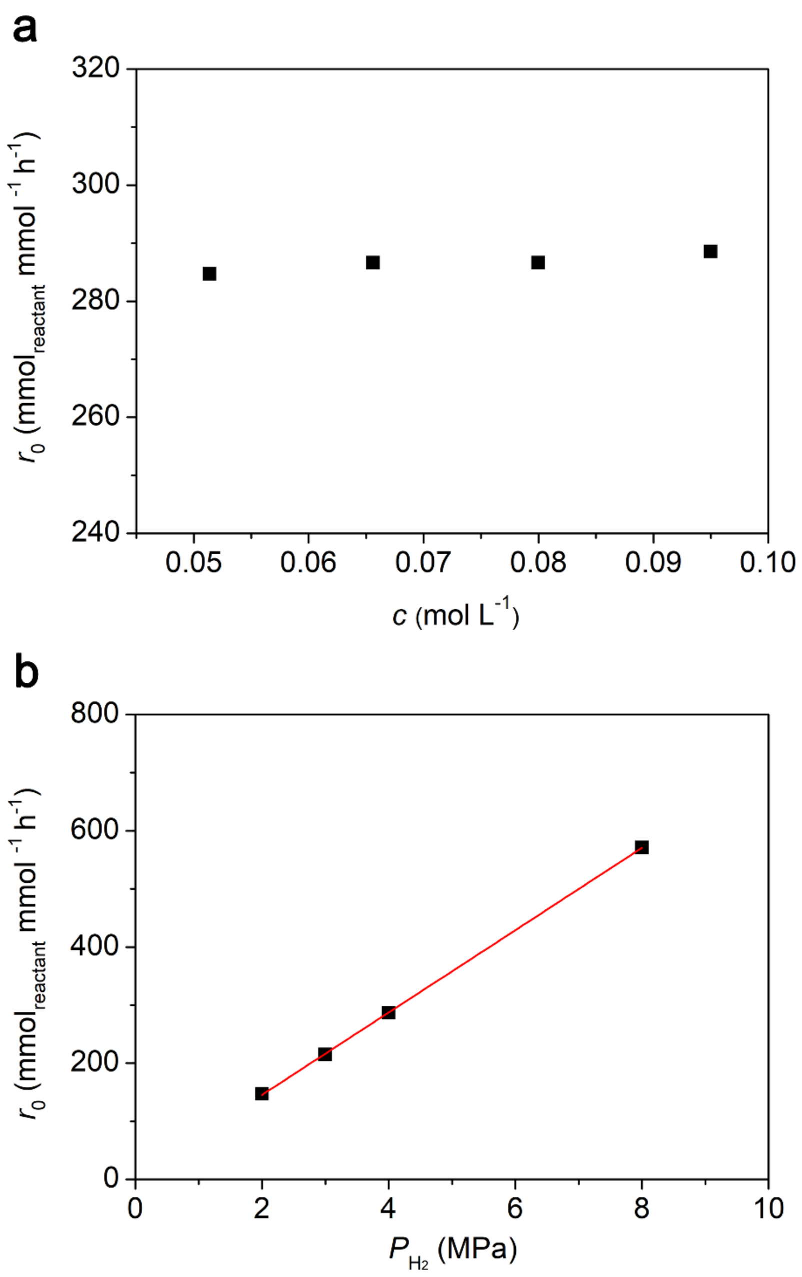

Supplementary Figure 19. Effect of (a) concentration of 3-nitrostyrene and (b) H_2 pressure on the reaction rate for the selective hydrogenation of 3-NS over C-Au-2.4/OMC. Reaction conditions: 20 mg of C-Au-2.4/OMC, 5 mL of ethanol, 140 °C, 800 rpm.

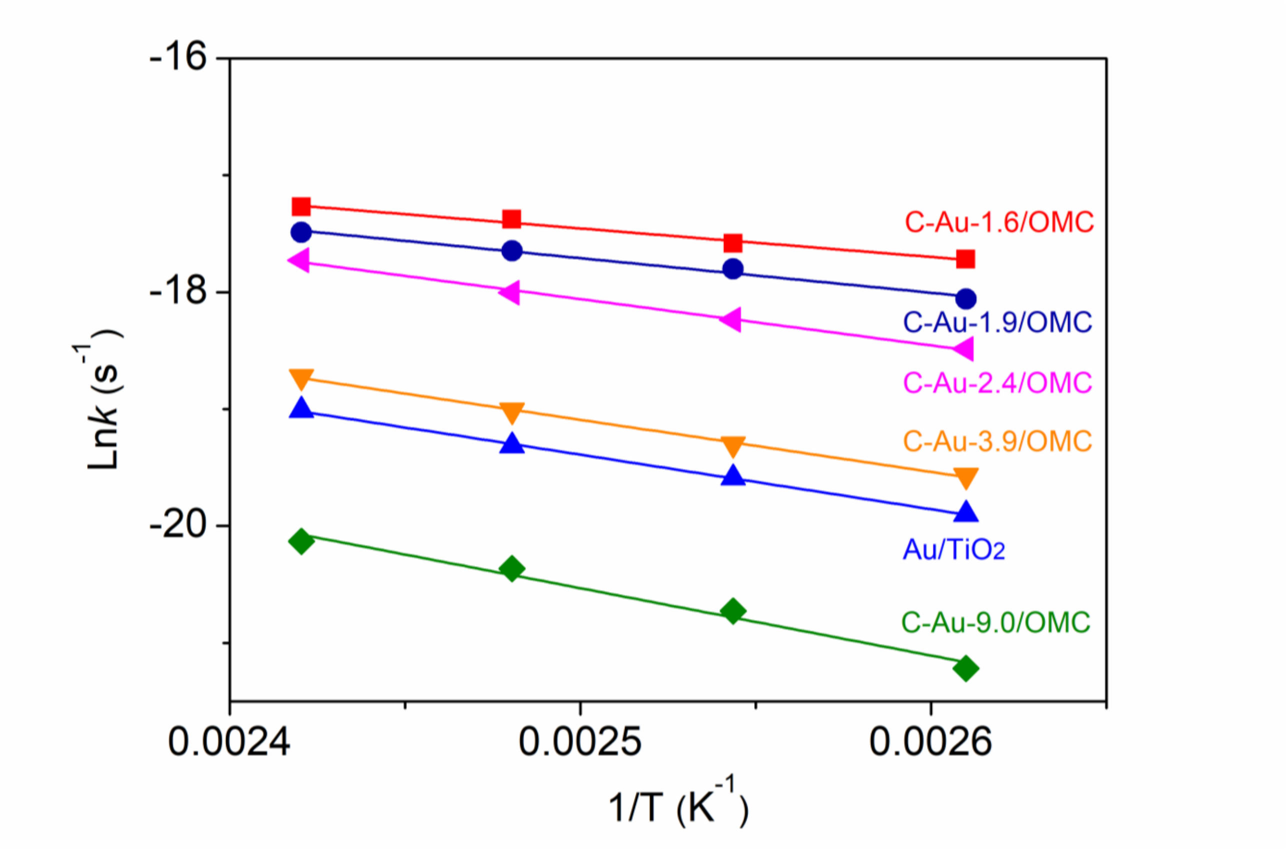

Supplementary Figure 20. Arrhenius plots for the hydrogenation of 3-NS over C-Au nanocatalysts with different Au nanoparticle sizes in the temperature range 110 - 140 °C.

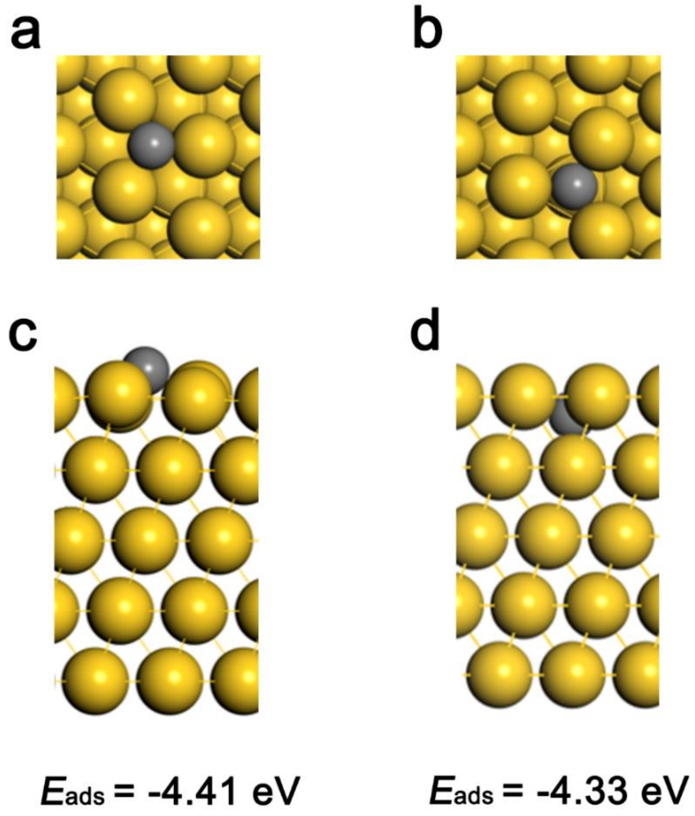

Supplementary Figure 21. Density functional theory (DFT) calculations. (a, b) Top and (c, d) side views of optimized configurations and adsorption energies of the (a, c) surface and (b, d) subsurface of C-modified Au(111).

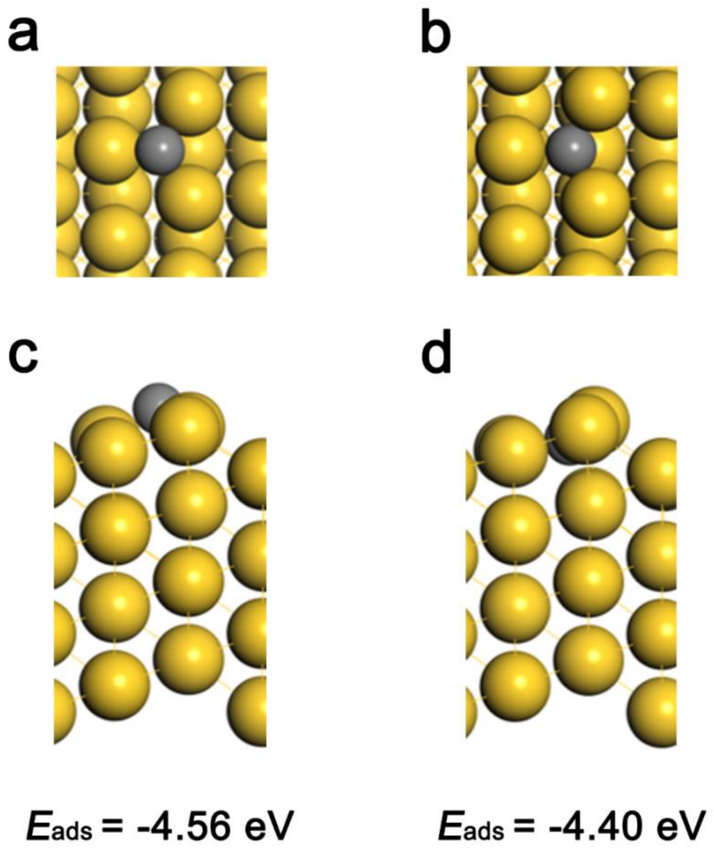

Supplementary Figure 22. DFT calculations. (a, b) Top and (c, d) side views of the optimized configurations and adsorption energies of the (a, c) surface and (b, d) subsurface of C-modified Au(211).

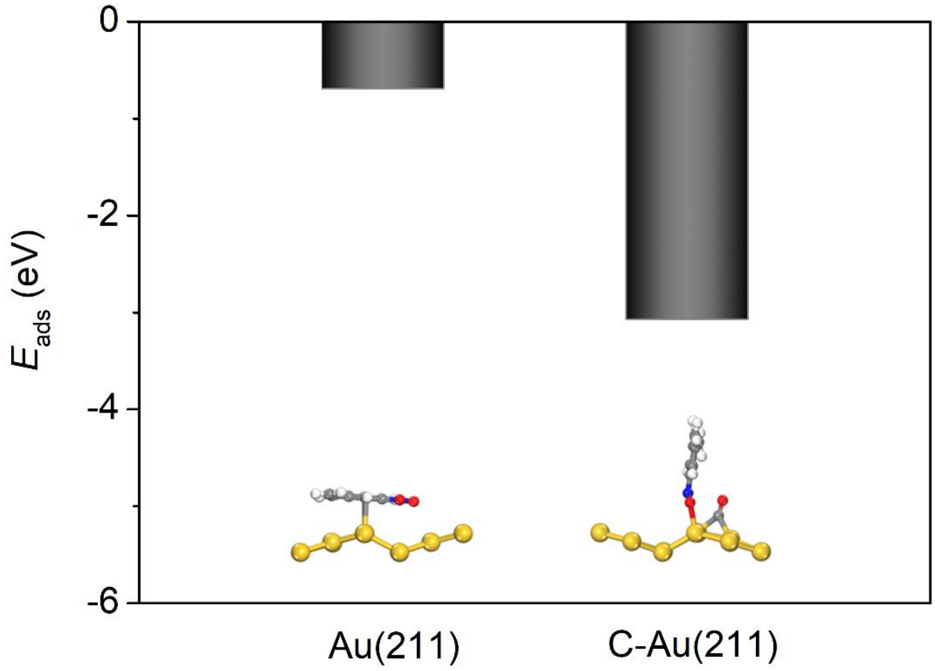

Supplementary Figure 23. Adsorption configuration and corresponding adsorption energies of 3-NS on clean Au(211) and C-modified Au(211) surfaces.

Supplementary Table 1. Structural properties of the Au nanocatalysts.

Sample	Au content[a] (wt%)	$d(111)$[b] (nm)	S_{BET} ($m^2\ g^{-1}$)	V_t ($cm^3\ g^{-1}$)	D_{metal}[c] (nm)
C-Au-1.6-OMC	0.64	0.239	469	0.47	1.6
C-Au-1.9/OMC	0.80	-	507	0.50	1.9
C-Au-2.4/OMC	0.92	0.238	483	0.50	2.4
C-Au-3.9/OMC	0.92	0.236	587	0.46	3.9
C-Au-9.0/OMC	0.92	-	521	0.51	9.0
Au/TiO_2	0.96	-	54	0.24	4.1
Au/TiO_2-R[d]	0.90	-	41	0.19	8.0
Au/SiO_2	1.07	-	294	1.15	5.8
Au/C	0.89	-	731	0.66	6.3
Au-2.4/OMC-R10[e]	0.91	-	462	0.48	2.4
OMC	-	-	594	0.56	-

[a] Au contents measured by inductively coupled plasma-atomic emission spectrometry (ICP-AES);
[b] *d*-spacing of Au(111) calculated from the spherical aberration corrected-STEM (AC-STEM) images;
[c] Particle size estimated from the TEM images;
[d] The Au/TiO_2 catalyst reused five times;
[e] The C-Au-2.4/OMC catalyst reused ten times.

Supplementary Table 2. Calculation of the *d*-electron gain of the Au nanocatalysts.

Sample	ΔA_3[b] (eV cm^{-1})	ΔA_2[b] (eV cm^{-1})	h_T[c]	$h_{5/2}$[d]	$h_{3/2}$[d]	$\Delta h_{5/2}$[e]	$\Delta h_{3/2}$[e]	Δh_T[f]
C-Au-1.6/OMC	-4099	-1608	0.209	0.134	0.056	-0.146	-0.064	-0.192
C-Au-1.9/OMC	-3516	-1391	0.236	0.155	0.065	-0.125	-0.055	-0.165
C-Au-2.4/OMC	-2919	-1168	0.264	0.176	0.073	-0.104	-0.047	-0.137
C-Au-3.9/OMC	-2446	-991	0.285	0.193	0.080	-0.087	-0.040	-0.116
C-Au-9.0/OMC	-1255	-547	0.340	0.236	0.098	-0.044	-0.022	-0.061
Au/TiO_2	-1819	-757	0.314	0.216	0.090	-0.064	-0.030	-0.087
Au/SiO_2	-89	-111	0.394	0.277	0.116	-0.003	-0.004	-0.007
Au/C	-164	-139	0.391	0.275	0.114	-0.005	-0.006	-0.010
Au foil[a]	-	-	0.401	0.283	0.118	-	-	-

[a] Values from Reference 1.
[b] ΔA_3 and ΔA_2 are the difference in the area under the L_3 and L_2 white line between NPs and bulk Au.
[c] h_T is total number of unoccupied *d* states.
[d] $h_{5/2}$ and $h_{3/2}$ are *d*-hole counts in the $d_{5/2}$ and $d_{3/2}$ orbitals.
[e] $\Delta h_{5/2}$ and $\Delta h_{3/2}$ are the change of *d*-hole counts in the $d_{5/2}$ and $d_{3/2}$ orbitals.
[f] Δh_T is the change in the total number of unoccupied *d* states.

The areas of different samples were determined using the part of the XANES spectrum that extended from 10 eV below the edge to 15 eV above the edge using Simpson's method. The fractional change in the number of *d* band vacancies (unoccupied *d* states) from those of the bulk Au, f_d, was defined as

$$f_d=\frac{\Delta h_T}{(h_T)_{\text{bulk Au}}}=(\Delta A_3+1.11\Delta A_2)/(A_{3r}+1.11A_{2r})_{\text{bulk Au}} \quad (1)$$

where $\Delta h_T=(h_T)_{\text{sample}}-(h_T)_{\text{bulk Au}}$.

$$A_{3r}=\rho\sigma_3\frac{h_{5/2}+h_{3/2}}{h_{5/2}}\left(A'_{3r}-A'_{2r}\right) \quad (2)$$

$$A_{2r}=\rho\sigma_2\frac{h_{3/2}}{h_{5/2}}\left(A'_{3r}-A'_{2r}\right) \quad (3)$$

$$\Delta A_3=\rho\sigma_3\left(A'_3-A'_{3r}\right) \quad (4)$$

$$\Delta A_2=\rho\sigma_2\left(A'_2-A'_{2r}\right) \quad (5)$$

where A'_{3r} and A'_{2r} are the integrated areas of bulk Au, A_{2r} and A_{3r} are the normalized area of bulk Au, A'_3 and A'_2 are the integrated areas for the samples. The area A_{3r} was found by first calculating the difference between the areas under the L_3 X-ray absorption edges over the range -10 to 40 eV. Second, this difference in area was then multiplied by the ratio $(h_{5/2} + h_{3/2})/h_{5/2}$ to approximately correct for the white-line weight at the L_2 X-ray absorption edge. The areas are normalized by multiplying by σ and ρ where σ is the X-ray absorption cross section at the edge jump and ρ is the density of the absorption material. Values of 107.7 and 57.3 cm^2 g^{-1} were used for the absorption cross sections at the Au L_3 and L_2 absorption edges. In terms of these normalized values and in units of eV cm^{-1}, A_{3r} = 1.064×10^4, A_{2r} = 1.67×10^3.

Changes in the number of vacancies in the $d_{5/2}$, $d_{3/2}$ states and ΔA_2 were calculated using the following expressions:

$$\Delta h_{5/2}=(2.25\Delta A_3\text{-}0.5\Delta A_2)/C \quad (6)$$

$$\Delta h_{3/2}=3\Delta A_2/C \quad (7)$$

$$\frac{h_{5/2}}{h_{3/2}}=2.4 \quad (8)$$

where C is the constant characteristic of the absorption (C = 75213 eV $cm^{-1})^2$.

The h_T value was calculated using the following equation [re-arrangement of Eq. (1)]:

$$h_T=\left(1+f_d\right)(h_T)_{\text{bulk Au}} \quad (9)$$

Supplementary Table 3. Synthesis conditions for C-Au/OMC with different sizes of Au nanoparticles.

Catalyst	MPTMS (g)	$HAuCl_4$[a] (mL)	Carbonization temperature (°C)
C-Au-1.6/OMC	0.128	1.1	600
C-Au-1.9/OMC	0.128	1.5	600
C-Au-2.4/OMC	0.096	1.5	700
C-Au-3.9/OMC	0.064	1.5	700
C-Au-9.0/OMC	0.015	1.5	700

[a] Volume of $HAuCl_4$ added in the reaction with a gold concentration in water of 24.3 mmol L^{-1};

Supplementary Table 4. Convergences of C adsorption on Au(111) surface with respect to the parameters used for the DFT calculations.

K-points tests

K-points	3×3×1	5×5×1	7×7×1	9×9×1
$E_{ads, C}$ (eV)	-4.97	-4.40	-4.55	-4.65

Cut-off energy tests

Cut-off energy (eV)	350	400	425	450
$E_{ads, C}$ (eV)	-4.41	-4.40	-4.40	-4.40

Supercell tests

Supercell	2×2	3×3
$E_{ads, C}$ (eV)	-4.35	-4.40

Vacuum layer tests

Vacuum layer (Å)	7.0	12.0	17.0
$E_{ads, C}$ (eV)	-4.40	-4.40	-4.40

Ionic relaxation criterion tests

EDIFFG (eV/Å)	0.03	0.025	0.020
$E_{ads, C}$ (eV)	-4.40	-4.40	-4.40

Electronic relaxation criterion tests

EDIFF (eV)	10^{-5}	10^{-6}	10^{-7}
$E_{ads, C}$ (eV)	-4.40	-4.40	-4.40

Atomic layer tests

Atomic layers	3	5	7
$E_{ads, C}$ (eV)	-4.40	-4.40	-4.01

Supplementary Table 5. Carbon atom at different sites of Au(111) surface and subsurface.

	Location	E_{ads} (eV)
Surface C	top site	-2.34
	fcc threefold hollow site	-4.41
	hcp threefold hollow site	-4.29
Subsurface C	tetrahedral interstice	-4.33
	octahedral interstice	-3.91

Supplementary Table 6. Carbon atom at different sites of Au(211) surface and subsurface.

	Location	E_{ads} (eV)
Surface C	Low *fcc*	-4.11
	Low *hcp*	-4.11
	High *fcc*	-4.42
	High *hcp*	-4.56
	Hollow	-4.53
	Bridge	-4.12
Subsurface C	Low octahedral	-3.86
	High octahedral	-3.65
	Step octahedral	-3.50
	Low tetrahedral	-4.26
	High tetrahedral	-4.40
	Step tetrahedral	-3.94

Supplementary Table 7. Adsorption energies of 3-NS on Au(111) and C-Au(111) surfaces.

	Configuration		E_{ads} (eV)
Au(111)	O_bridge	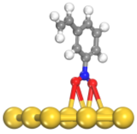	-0.51
	Parallel	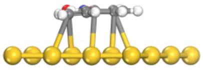	-0.81
	Perpendicular	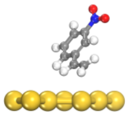	-0.41
C-Au(111)	C_top	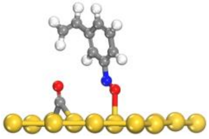	-3.04
	O_bridge	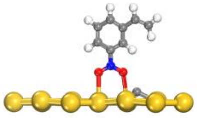	-0.41
	Parallel	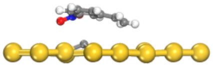	-0.78
	Perpendicular	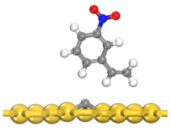	-0.50

Supplementary Table 8. Adsorption energies of 3-NS on Au(211) and C-Au(211) surfaces.

	Configuration		E_{ads} (eV)
Au(211)	O_bridge	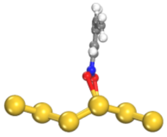	-0.28
	Parallel	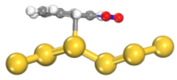	-0.69
	Perpendicular	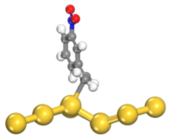	-0.29
C-Au(211)	C_top	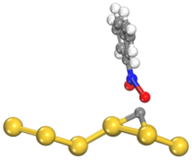	-2.83
	O_bridge	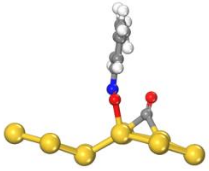	-3.07
	Parallel	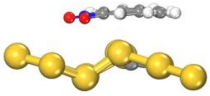	-0.51
	Perpendicular	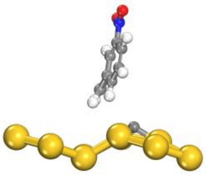	-0.23

Supplementary Methods

Kinetics

Elemental Reaction Steps for Hydrogenating Nitroaromatic Compounds on C-Au/OMC Catalysts:

The following elemental steps are involved during the hydrogenation of the nitroaromatic compound on C-Au/OMC catalysts:

$$H_2 + 2^* \rightarrow 2H^* \quad (10)$$

$$\text{3-NS} + {}^* \leftrightarrow \text{3-NS}^* \quad (11)$$

$$2H^* + \text{3-NS}^* \leftrightarrow \text{Products} \quad (12)$$

where the adsorption/dissociation of H_2 is the rate controlling step of the whole process, while the remaining steps are in equilibrium (* represents a free active site on the surface of the support).

Working at the initial reaction rates, it can be assumed that the concentration of all the reaction products (either in the liquid phase, or adsorbed) is negligible, so we have simplified the overall reaction scheme by omitting some intermediate reaction steps between the adsorption of the reactants and the formation of the final product (3-vinylaniline).

$$r = \frac{k_{H_2} \cdot P_{H_2}}{(1 + K_{\text{3-NS}} \cdot C_{\text{3-NS}})^2} \quad (13)$$

$$\sqrt{\frac{P_{H_2}}{r}} = \frac{1}{\sqrt{k_{H_2}}} + \frac{K_{\text{3-NS}}}{\sqrt{k_{H_2}}} \cdot C_{\text{3-NS}} \quad (14)$$

where r is the rate of reaction, P_{H_2} is the pressure of hydrogen, $K_{\text{3-NS}}$ is the adsorption constant of 3-nitrostyrene in the equilibrium and $C_{\text{3-NS}}$ is concentration of 3-nitrostyrene.

Exposed Surface Atom Dispersion of C-Au/OMC Catalysts:

We assumed that the value for the exposed surface atom dispersion (τ) calculated from equations (15) - (18), which was based on the similarities of the truncated octahedron shape of the nanoparticles (as seen in the AC-STEM image), was accurate[3].

$$d_{\text{particle}} = 1.105 \times N_T^{1/3} \times d_{\text{Au}} \quad (15)$$

$$N_T = 16m^3 - 33m^2 + 24m - 6 \quad (16)$$

$$N_S = 30m^2 - 60m + 32 \quad (17)$$

$$\tau = N_S / N_T \quad (18)$$

where d_{particle} is the diameter of the particle, N_T is the total number of atoms in each particle, N_S is the number of surface atoms, and m is the number of atoms lying on an equivalent edge (corner atoms included). Note that the diameter of a Au (d_{Au}) atom is 0.2884 nm.

TOF for Hydrogenating Nitroaromatic Compounds on C-Au/OMC Catalysts:

The TOF for each catalyst was calculated on the basis of the estimated number of exposed Au atoms. This value was calculated at less than 25% conversion,

$$\text{TOF} = \frac{n_{3-\text{NS}} X}{n_{\text{Au}} t \tau} \quad (19)$$

where X is the conversion, t is the reaction time.

Entropy of activation for Hydrogenating Nitroaromatic Compounds on C-Au/OMC Catalysts:

Activation energies E_a were calculated from the Arrhenius equation:

$$k = A\exp\left(\frac{-E_a}{RT}\right) \tag{20}$$

$$\ln k = \ln A - \frac{E_a}{RT} \tag{21}$$

where k is the reaction rate constant, A is the pre-exponential factor, R is the universal gas constant and T is the reaction temperature.

The entropy of activation is determined as follows:

$$\mathrm{A} = \frac{k_B T}{h} e^n \left(\frac{p^\theta}{RT}\right)^{1-n} \exp\left[\frac{\Delta S^{0*}(p^\theta)}{R}\right] \tag{22}$$

where k_B, h, ΔS^{0*}, and p^θ are the Boltzmann constant, Planck constant, entropy of activation, and standard pressure, respectively.

Taking into account the approximate first-order reaction kinetics for 3-nitrostyrene. The entropy change in the activation step of the chemical reaction is closely related to the thermodynamics of the rate constant, which results in the following equation:

$$\Delta S^{0*} = R\ln\left(\frac{Ah}{k_B T e \tau}\right) \tag{23}$$

Computational details

All the DFT calculations were carried out by the Vienna Ab-initio Simulation Package (VASP)[4-7], where the GGA-PBE[8] density functional was employed. A projected augmented wave method (PAW)[9,10] with a cut-off energy of 400 eV was used to describe the interaction between the ionic core and valence electrons. A 5×5×1 gamma-centered k-point mesh was used for the clean surface, surface carbon and subsurface carbon modified ones, which were modeled by five-layer Au(111) and Au(211) surfaces with p(2×2) supercells, and electronic occupancies were determined by a first order Methfessel-Paxton scheme[11] with a smearing width of 0.2 eV. The top three layers were relaxed, while the bottom two were fixed at the bulk lattice positions. A 12 Å of vacuum layer was set between the periodically repeated slabs to avoid inter-slab interactions. The criteria for the convergence of electronic, and ionic relaxations were set to be 10^{-5} eV and 0.03 eV $Å^{-1}$, respectively. All the parameters were optimized with respect to the adsorption energy of carbon atom on the clean Au(111) surface (Supplementary Table 4). The locations of carbon atoms on the surface and subsurface were comparatively investigated for clean and C-modified Au(111) and Au(211) surfaces and subsurfaces (Supplementary Tables 5 and 6). The isolated molecules and atoms were optimized in a box of 20 Å × 20 Å × 20 Å with the same criterion as those for geometric optimization of the Au surfaces. The adsorption energies were calculated by $E_{ads} = E_{adsorbate/surface} - E_{surface} - E_{adsorbate}$, where $E_{adsorbate/surface}$, $E_{surface}$ and $E_{adsorbate}$ are respectively the total energy of a surface covered with an adsorbate, the clean surface slab, and an isolated adsorbate. The transition states of hydrogen dissociation on the surfaces were located by the dimer method[12] where the criteria

for the convergence of electronic, and ionic relaxations were set to be 10^{-6} eV and 0.025 eV $Å^{-1}$, respectively. The geometrical structures for the transition states were confirmed by subsequent vibrational calculations carried out using the numerical finite difference method, which generated only one imaginary frequency for a specific transition state. The zero-point energy correction was included in the calculation for the energy barrier of H_2 dissociation. The adsorption configurations and energies for 3-NS on the studied surfaces are compiled in Supplementary Tables 7 and 8.

Supplementary References

1. Ohyama, J. et al. In situ Au L_3 and L_2 edge XANES spectral analysis during growth of thiol protected gold nanoparticles for the study on particle size dependent electronic properties. *Chem. Phys. Lett.* **507**, 105-110 (2011).
2. Zhang, P. & Sham, T. K. Tuning the electronic behavior of Au nanoparticles with capping molecules. *Appl. Phys. Lett.* **81**, 736-738 (2002).
3. Fu, W. et al. Kinetics-assisted discrimination of active sites in Ru catalyzed hydrolytic dehydrogenation of ammonia borane. *React. Chem. Eng.* **4**, 316-322 (2019).
4. Kresse, G. & Hafner, J. Ab initio molecular dynamics for liquid metals. *Phys. Rev. B* **47**, 558-561 (1993).
5. Kresse, G. & Hafner, J. Ab initio molecular-dynamics simulation of the liquid-metal-amorphous-semiconductor transition in germanium. *Phys. Rev. B* **49**, 14251-14269 (1994).
6. Kresse, G. & Furthmüller, J. Efficiency of ab-initio total energy calculations for metals and semiconductors using a plane-wave basis set. *Comput. Mater. Sci.* **6**, 15-50 (1996).
7. Kresse, G. & Furthmüller, J. Efficient iterative schemes for ab initio total-energy calculations using a plane-wave basis set. *Phys. Rev. B* **54**, 11169-11186 (1996).
8. Perdew, J. P., Burke, K. & Ernzerhof, M. Generalized gradient approximation made simple. *Phys. Rev. Lett.* **77**, 3865-3868 (1996).
9. Blöchl, P. E. Projector augmented-wave method. *Phys. Rev. B* **50**, 17953-17979 (1994).
10. Kresse, G. & Joubert, D. From ultrasoft pseudopotentials to the projector augmented-wave method. *Phys. Rev. B* **59**, 1758-1775 (1999).
11. Methfessel, M. & Paxton, A. T. High-precision sampling for Brillouin-zone integration in metals. *Phys. Rev. B* **40**, 3616-3621 (1989).
12. Henkelman, G. & Jónsson, H. A dimer method for finding saddle points on high dimensional potential surfaces using only first derivatives. *J. Chem. Phys.* **111**, 7010-7022 (1999).
